# Method for cryopreservation of trigeminal ganglion for establishing primary cultures of neurons and glia

**DOI:** 10.1016/j.jneumeth.2023.110034

**Published:** 2023-12-10

**Authors:** Sophia R. Antonopoulos, Mikayla Scharnhorst, Nicole Nalley, Paul L. Durham

**Affiliations:** Missouri State University, Jordan Valley Innovation Center/Department of Biology, Springfield, MO 65806, USA

**Keywords:** Trigeminal ganglion, Cryopreservation, Satellite glia, Schwann cells, Immunocytochemistry, Calcium imaging

## Abstract

**Background:**

Primary neuronal cultures are used to elucidate cellular and molecular mechanisms involved in disease pathology and modulation by pharmaceuticals and nutraceuticals, and to identify novel therapeutic targets. However, preparation of primary neuronal cultures from rodent embryos is labor-intensive, and it can be difficult to produce high-quality consistent cultures. To overcome these issues, cryopreservation can be used to obtain standardized, high-quality stocks of neuronal cultures.

**New method:**

In this study, we present a simplified cryopreservation method for rodent primary trigeminal ganglion neurons and glia from Sprague-Dawley neonates, using a 90:10 (v/v) fetal bovine serum/dimethyl sulfoxide cell freezing medium.

**Results:**

Cryopreserved trigeminal ganglion cells stored for up to one year in liquid nitrogen exhibited similar neuronal and glial cell morphology to fresh cultures and retained high cell viability. Proteins implicated in inflammation and pain signaling were expressed in agreement with the reported subcellular localization. Additionally, both neurons and glial cells exhibited an increase in intracellular calcium levels in response to a depolarizing stimulus. Cryopreserved cells were also transiently transfected with reporter genes.

**Comparison with existing methods:**

Our method is simple, does not require special reagents or equipment, will save time and money, increase flexibility in study design, and produce consistent cultures.

**Conclusions:**

This method for the preparation and cryopreservation of trigeminal ganglia results in primary cultures of neurons and glia similar in viability and morphology to fresh preparations that could be utilized for biochemical, cellular, and molecular studies, increase reproducibility, and save laboratory resources.

## 1. Introduction

The trigeminal ganglion is implicated in the underlying pathology of orofacial pain conditions including migraine, temporomandibular disorders (TMD), and trigeminal neuralgia (Goadsby et al., 2017; Chichorro et al., 2017). In addition, inflammation and pain associated with oral structures such as teeth and gums are also mediated via trigeminal ganglion nerves (Martinez-Garcia et al., 2019). The trigeminal ganglion has three main nerve branches including the ophthalmic (V1), maxillary (V2), and mandibular (V3) (Shankland, 2001a, 2001b, 2001c). These branches provide sensory innervation to most of the head and face and are the pathways responsible for transmission of painful stimuli from peripheral tissues to the central nervous system. The cell bodies of type Aδ and C fiber neurons are surrounded by satellite glial cells (Durham and Garrett, 2010). Together they form functional units that facilitate neuron-glia interactions. In response to tissue injury or ischemic events, activation of trigeminal ganglion Aδ and C fibers promote an inflammatory response, initiate pain signaling, and enhance neuron-glia communication associated with peripheral sensitization (Durham, 2016; Messlinger et al., 2020). The dysfunction of glial cells, which has been termed gliopathy, is associated with persistent neuronal sensitization and enhanced nociception (Ji et al., 2013). Within the trigeminal ganglion, the major glial cell types are satellite glia and Schwann cells. Satellite glia, which are found in close association with neuronal cell bodies located in the ganglion, modulate the excitability state of nociceptive neurons, and help to promote and maintain peripheral sensitization via paracrine signaling and formation of gap junctions that metabolically and ionically couple neurons and glia. Schwann cells are another ganglion glial cell that are classified by two types: myelinating Schwann cells that wrap Aβ and Aδ in a myelin sheath, and the non-myelinating Schwann cells that encompass C fibers to form a Remak bundle (Jessen and Mirsky, 2016). Under normal physiological conditions, both types of Schwann cells can support, nourish, and insulate axons. Together, both types of glia, satellite glia and Schwann cells, function as neuromodulators to regulate the excitability state of trigeminal ganglion neurons (Messlinger et al., 2020).

Peripheral sensitization and activation of trigeminal ganglion neurons and glial cells involves multiple signal transduction pathways known to promote an inflammatory response and enhance pain signaling from the ganglion to the spinal cord (Malick and Burstein, 2000; Bernstein and Burstein, 2012). Calcitonin gene-related peptide (CGRP) is a neuropeptide synthesized and released by trigeminal neurons; elevated levels are implicated in the pathology of migraine and TMD (Blumenfeld et al., 2021; Sato et al., 2004; Ambalavanar and Dessem, 2009). CGRP binding to its receptors on neurons and glial cells leads to increases in intracellular levels of the secondary messenger cAMP and activation of protein kinase A (PKA) (Sun et al., 2004; Cady et al., 2011; Cornelison et al., 2016). PKA promotes a sensitized state via phosphorylation of ion channels and receptors in neurons and stimulating synthesis and release of cytokines and other pro-inflammatory molecules (Nie et al., 2022). Elevated levels of CGRP and PKA are associated with increased expression of the active forms of the mitogen-activated protein (MAP) kinases ERK, JNK, and p38 in neurons and glial cells that promote an inflammatory response and enhanced pain signaling (Cady et al., 2011; Schaeffer et al., 2003; Vause and Durham, 2009, 2010). Other proteins, including the MAP kinase phosphatase MKP-1 and GABAergic proteins, are involved in restoring and maintaining homeostasis by inhibiting activation of neurons and glial cells. MKP-1 causes inactivation of the MAP kinases and suppresses the development and maintenance of an excitable state of neurons and glia (Owens and Keyse, 2007). GABA is an inhibitory neurotransmitter synthesized by the enzymes GAD 65/67 (Lee et al., 2019). GABA binding to GABAA and GABAB receptors on neurons and glial cells in the ganglion suppresses neuronal and glial activation (Hayasaki et al., 2006).

Primary cultures of neuronal and glial cells from sensory ganglion provide a valuable *in vitro* model to investigate biochemical, cellular, and molecular changes in specific cell types in response to various stimuli, pharmaceuticals, and nutraceuticals (Nie et al., 2022; Kunioku et al., 2023; Moore et al., 2023; Yazaki et al., 2022; Verissimo et al., 2022; Takacs-Lovasz et al., 2022; Belinskaia et al., 2022; Inoue et al., 2021). Specifically, primary cultures of trigeminal ganglia have been utilized for elucidating and understanding pathological and therapeutic processes in neurons and glial cells that have aided in our understanding of orofacial pain diseases (Durham and Russo, 1999, 2003; Durham and Cady, 2004; Durham et al., 2004; Bowen et al., 2006; Bellamy et al., 2006; Durham et al., 2006). Compared to *in vivo* studies, the use of primary cultures provides a simpler and more economical method for determining pathological and therapeutic mechanisms to identify novel molecular targets. However, the continual establishment of primary cultures is time-consuming, can be technically challenging, yields inconsistencies in cell density and function, and requires many animals. Alternatively, the use of cryopreservation allows for flexibility in planning experiments since this method can slow or stop metabolic and cellular processes to maintain the viability and function of cells for extended periods of time. The goal of this study was to develop an efficient and reliable method for long-term cryopreservation of trigeminal ganglion from neonatal Sprague Dawley rats to allow for greater flexibility in experimental design and cost savings when establishing primary neuronal and/or glial cultures for biochemical, cellular, and molecular studies involving toxicity, protein expression, calcium ion imaging, and gene expression.

## 2. Materials and methods

### 2.1. Animals

Male and female neonatal Sprague-Dawley 3–5-day old pups were utilized for establishing primary cultures of the trigeminal ganglion. All experiments were approved by the Institutional Animal Care and Use Committee at Missouri State University, and conducted in accordance with institutional, ARRIVE, and NIH guidelines (Protocol 2022-08 approved on April 29, 2022; Animal Welfare Assurance Number D16-00033). A concerted effort was made to minimize the number of animals used for this study. Female pregnant adult Sprague-Dawley rats were purchased from Missouri State University’s internal breeding colony (Springfield, MO). Animals were housed in clear, plastic cages with unrestricted access to food and water. Animal holding rooms were on a 12-hour light/dark cycle and maintained at a constant temperature of 22–24 °C. The animal’s overall health was monitored daily by vivarium staff. No adverse events were associated with this study.

### 2.2. Establishing primary cultures of trigeminal ganglia

Primary cultures of trigeminal ganglia were established based on prior studies from our laboratory (Durham et al., 1997; Durham and Masterson, 2013; Antonopoulos and Durham, 2022). Trigeminal ganglia were obtained from neonatal 3–5-day old rat pups and placed in 4 °C HEPES buffered L-15 media (pH 7.4), referred to as plating media, during the duration of dissections. Following dissections, tissues were placed in room temperature plating media with Dispase II (Sigma Aldrich, St. Louis, MO) and RQ1 DNase (Promega, Madison, WI), and incubated at 37 °C for 30 min while being constantly rotated at 15 RPM to enzymatically digest connective tissue and promote cell dissociation. Following incubation, tissues were centrifuged at 500 RPM for 2–3 min, the resulting supernatant was decanted, and tissues resuspended in 5 mL of plating media. Vigorous mechanical trituration was performed to further dissociate the tissues. The supernatant containing cells was transferred to a sterile 15 mL tube, and the dissociation step was repeated. The final 10 mL of cell-containing supernatant was centrifuged at 1300 RPM for 3 min to pellet dissociated neurons and glial cells. For cryopreservation, cells were resuspended in a 37 °C solution of 90% fetal bovine serum (FBS, Atlanta Biologicals, Norcross, GA), and 10% dimethyl sulfoxide (DMSO, Sigma), and aliquoted into cryogenic vials for storage. Vials were slowly frozen using a Mr. Frosty freezing container (ThermoFisher Scientific, Waltham, MA) placed inside of a −80 °C freezer overnight. The following day, vials were quickly transferred to a liquid nitrogen Dewar where they remained in long-term storage for approximately one year. For plating of fresh cell culture preparations, the cell pellet was resuspended in a 37 °C L-15 medium containing 10% FBS (Atlanta Biologicals), 50 mM glucose (Sigma-Aldrich), 250 mM ascorbic acid (Sigma-Aldrich), 8 mM glutathione (Sigma-Aldrich), 2 mM glutamine (Sigma-Aldrich), 10 ng/mL mouse 2.5 S nerve growth factor (Alomone Laboratories, Jerusalem, Israel), an antibiotic mixture of penicillin (100 units/mL) and streptomycin (100 μg/mL, Sigma-Aldrich), and the antimycotic amphotericin B (2.5 mg/mL, Sigma-Aldrich); this media will be referred to as TG complete medium. To begin an experiment using cryopreserved cells, vials of frozen cells were gradually thawed by addition of 37 °C TG complete medium. One vial consisting of 0.5 mL of cryopreservation solution was thawed in 5–10 mL of warm TG complete medium to immediately dilute the DMSO, to minimize the loss of cell viability. For immunocytochemistry, live/dead assay, and calcium imaging, fresh or cryopreserved cells were plated on 12 mm poly-D-lysine coated glass coverslips (Electron Microscopy Sciences, Hatfield, PA) at a density of ~1 ganglion (~34,000 cells) per 24-well plate (Greiner Bio-One, Monroe, NC) in 500 μL of TG complete medium per well. For transfection studies, a density of ~2 ganglia per well was used and plated on 24-well tissue culture treated plates (Corning Incorporated-Life Sciences, Durham, NC) in 500 μL of TG complete medium. Cultures for all experiments were left to incubate at 37 °C in a humidified chamber at ambient CO_2_ levels. Cells were observed daily using a light microscope to look for any abnormal cell morphologies, low cell density, and pH of the media was monitored via the color indicator present in the L-15 media.

### 2.3. Characterization of cryopreserved cultures

Differential interphase contrast (DIC) images (200x) of overnight cultures stained with the nuclear fluorescent dye 4′,6-diamidino-2-phenylindole (DAPI, Vector Laboratories, Burlingame, CA) were acquired using a Zeiss Axiocam mRm camera (Carl Zeiss, Thornwood, NY) mounted on a Zeiss Imager Z1 fluorescent microscope to identify and quantify the types of cells based on their unique morphology (n = 5). The LIVE/DEAD Cell Imaging assay (Invitrogen, Waltham, MA) was used to assess the viability of glial cells and neurons following cryopreservation. Cells were plated at ~1 trigeminal ganglion per 24-well plate onto poly-D-lysine coated glass coverslips which corresponds to ~1500 cells per coverslip. Cells were allowed to grow in TG complete growth medium for a minimum of one day. The assay was performed following the manufacturer’s instructions. Briefly, the permeable green “live dye” and the cell impermeable red “dead” dye were mixed in a 1:1 ratio to create a 2x working solution. Up to four coverslips were removed from the 24 well plate and placed onto a glass microscope slide. Next, 100 μL of the 2x dye solution and 100 μL of phosphate-buffered saline (PBS) was added to each slide containing coverslips of cells and incubated at room temperature for 10–15 min to stain the cells. Stained cells were covered with a glass coverslip and imaged with a Zeiss Axiocam mRm camera mounted on a Zeiss Imager Z1 fluorescent microscope. A minimum of three images were captured of each coverslip and used for analysis. The number of live cells and dead cells were manually counted. Results are reported as the average number of live vs dead cells in the percentage of total cells ± standard error of the mean (n = 12, in triplicate). Fresh and cryopreserved cells were compared statistically via a Mann Whitney U test, and results were considered significant if *p* < 0.05.

### 2.4. Immunocytochemistry

Protein expression in neurons and glia was investigated using immunocytochemistry as described in prior studies (Vause and Durham, 2009; Antonopoulos and Durham, 2022). Primary cultures of cryopreserved ganglion were allowed to incubate for at least one day prior to fixation via incubation for 15 min in a 4% paraformaldehyde solution diluted in PBS. Cells were incubated for 20 min with a PBS solution containing 0.1% Triton X-100 (Sigma Aldrich) and 5% donkey serum (Jackson ImmunoResearch Laboratories, West Grove, PA) to block and permeabilize the cells. Cells were next incubated with primary antibodies diluted in 1% donkey serum and incubated overnight at 4 °C in a humidified chamber (Table 1). Cells were then incubated with Alexa-Fluor conjugated secondary antibodies (Jackson ImmunoResearch Laboratories) diluted 1:200 in PBS for 1 h at room temperature. Coverslips were rinsed with PBS prior to being placed on double frosted glass microscope slides (ThermoFisher Scientific). Vectashield anti-fade mounting medium containing the dye DAPI was added to each coverslip to stain the nucleus of each cell and to preserve the fluorescent signal in the cells. A rectangular glass coverslip was placed over each slide and secured using a clear coat of nail polish. Slides were stored at 4 °C until imaging. Images were taken at 200x magnification within two weeks of immunostaining using a Zeiss Axiocam mRm camera mounted on a Zeiss Imager Z1 fluorescent microscope to capture images of fluorescent staining as well as to identify cell morphology with DIC images. As a control, some slides were incubated with only secondary antibodies and no cell staining was visible. Neurite outgrowth was evaluated using Zen Software version 3.8 to measure the length of neuronal processes (μm).

### 2.5. Calcium imaging

To demonstrate functional ion channels in both neurons and glial cells, intracellular calcium levels were determined at basal levels and in response to stimulation with 60 mM potassium chloride (KCl) using the ratiometric fluorescent dye Fura-2 essentially as previously described (Bellamy et al., 2006; Durham and Masterson, 2013; Abbey et al., 2008; Masterson and Durham, 2010). Cryopreserved primary cultures were plated on poly-D-lysine coated glass coverslips at a density of approximately 1 trigeminal ganglion per 24-well plate and incubated overnight at 37 °C. The following day cells were rinsed with a standard HEPES-buffered saline (HBS, pH 7.4) solution two times, and then 500 μL of HBS was added to each well. Fura-2 AM ester (ThermoFisher Scientific) stock solution at a concentration of 1 mM was added to the well at 1:1000 for a final concentration of 1 μM in the well and cells incubated for 30 min at 37 °C for loading of the Fura-2 dye. After incubation, cells were rinsed twice with HBS and then incubated for 30 min at 37 °C before initiating calcium measurements. Using an Olympus IX83 microscope and Olympus CellSens Dimensions software version 4.1, (Evident Scientific, Tokyo, Japan) neurons and glial cells were clearly identified by their unique morphologies and size differences. Inside the 37 °C heated and humidified chamber, absorbance was measured at 340 and 380 nm every 20 s for 40 s to establish baseline measurements. After baseline measurements were established, 3 M KCl was added at a 1:50 dilution to the HBS for a final concentration of 60 mM, and ratiometric absorbance measurements immediately continued. After the addition of KCl, the change in absorbance was measured once every second for 20 s and then switched to measurements taken every 20 s for 80 s, for a total measurement time of 100 s after stimulation. Data are represented as graphs of the average ratio of F340/F380 nm wavelength values that corresponds to bound and unbound intracellular calcium levels in each of the four cell types: C fiber neurons, Aδ fiber neurons, satellite glial cells, and Schwann cells. Each experiment was performed a minimum of 5 times.

### 2.6. Transient transfection

Transient transfection of trigeminal ganglion primary cultures was performed similarly to prior published studies (Durham and Russo, 1999, 2003, 1998). Primary cells were thawed and plated at a density of ~2 ganglia per well (~68,000 cells) on 24-well tissue culture treated plates in 450 μL of TG complete growth media. Approximately 3 h after plating, cells were transiently transfected following the Lipofectamine 3000 Transfection Reagent protocol (L3000015, ThermoFisher Scientific). Lipofectamine was diluted in Opti-MEM, a serum free media in a ratio of 0.15 μL of Lipofectamine to 25 μL of media. Plasmid DNA containing the CMV-β-galactosidase and CMV-luciferase plasmids were mixed in a separate tube with the P3000 reagent, with 100 ng of each plasmid. A ratio of 0.2 μL of P3000 reagent to 100 ng of DNA was used per 25 μL of Opti-MEM media. The two solutions containing Lipofectamine and the P3000 plus plasmid DNA were combined in a 1:1 ratio, mixed well, and incubated for 10–15 min at room temperature to form a lipid-DNA complex. The lipid-DNA complex solution (25 μL) was directly added to each well containing cells. After overnight incubation at 37 °C, cells were rinsed with PBS and manually scraped from the wells. Cells were pelleted via centrifugation and resuspended in cell lysis buffer (Promega). Luciferase assay samples were measured by combining 10 μL of sample with 50 μL of the Luciferase Assay Substrate (E151A, Promega) in a clear 1.7 mL tube, briefly vortexed, and the relative light units measured in a luminometer (Berthold, Oak Ridge, TN) for 10 s. The β-galactosidase assay (Galacto-Light Plus ^™^ β-Galactosidase Reporter Gene Assay System, ThermoFisher Scientific) was performed by mixing 2.5 μL of sample with 50 μL of reaction buffer and incubated for 30–60 min. After incubation at room temperature, 75 μL of Accelerator-II was added before vortexing the sample. Relative light units were measured in the luminometer for 10 s. A standard Bradford protein quantification assay (Quick-Start Bradford Protein Assay, Bio-Rad, Hercules, CA) was performed, with absorbance measured at 595 nm on a microplate spectrophotometer. Protein levels for each individual well were calculated and used to normalize luciferase and β-galactosidase assay data. Transfection experiments were performed in duplicate for 4 individual experiments.

## 3. Results

### 3.1. Primary culture cell types and morphology

**Fig. 1.**
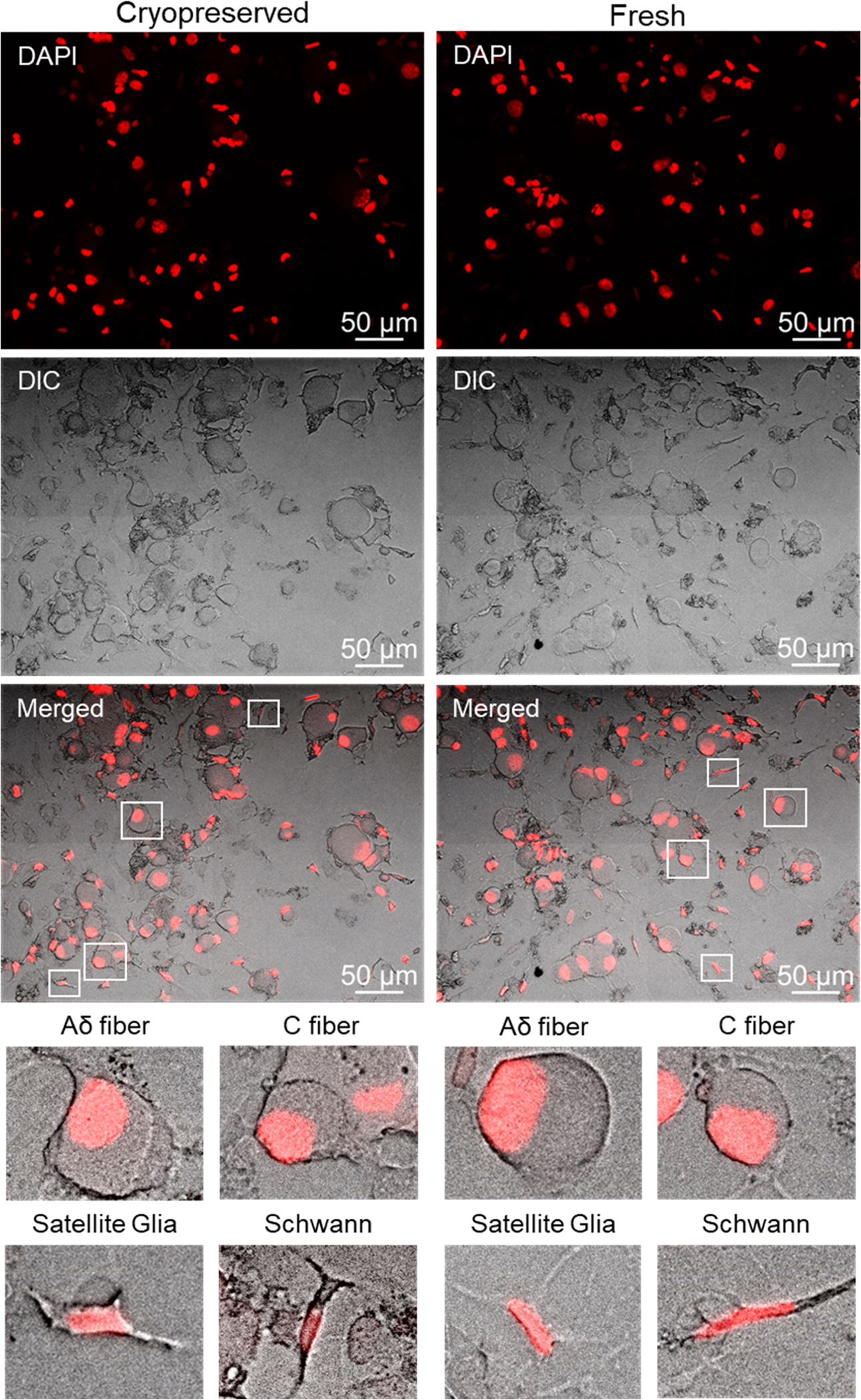
The cell types and morphology are similar in cryopreserved and fresh trigeminal ganglion primary cultures. DAPI-stained, DIC, and merged images are shown of cryopreserved and fresh primary cultures following overnight incubation. Enlarged images highlight the morphology and presence of each of the four cell types: Aδ fiber neurons, C fiber neurons, satellite glial cells, and Schwann cells.

Primary cultures from the trigeminal ganglion of rats that were established using cryopreserved cells exhibited identical cell types and similar morphology compared to fresh overnight cultures prepared the previous day (Fig. 1). The four main cell types present in trigeminal ganglion were observed in the cryopreserved cultures including C fiber neurons, Aδ fiber neurons, satellite glial cells, and Schwann cells. As expected, cryopreserved and fresh culture preparations showed similar numbers of each cell type (Table 2). Satellite glia and Schwann cells were more abundant than Aδ and C fiber neuronal cells in both types of cell preparations (n = 5 independent experiments done in triplicate). Cell types were readily identified based on the unique morphology and size of the cell body in phase contrast images and the associated DAPI-stained nuclei. Neurons were identified based on their rounded, large cell body with Aδ fiber neurons having a larger cell body (> 30 μm) than C fiber neurons (< 30 μm). Satellite glial cells were identified based on their small, elongated flat cell body, round nucleus, and multiple processes. In contrast, Schwann cells exhibit a bipolar morphology with an elongated nucleus and extended processes. The percentage and morphology of neuronal and glial cells of cryopreserved cultures was similar to that observed in fresh culture preparations.

### 3.2. Cell viability after cryopreservation

**Fig. 2.**
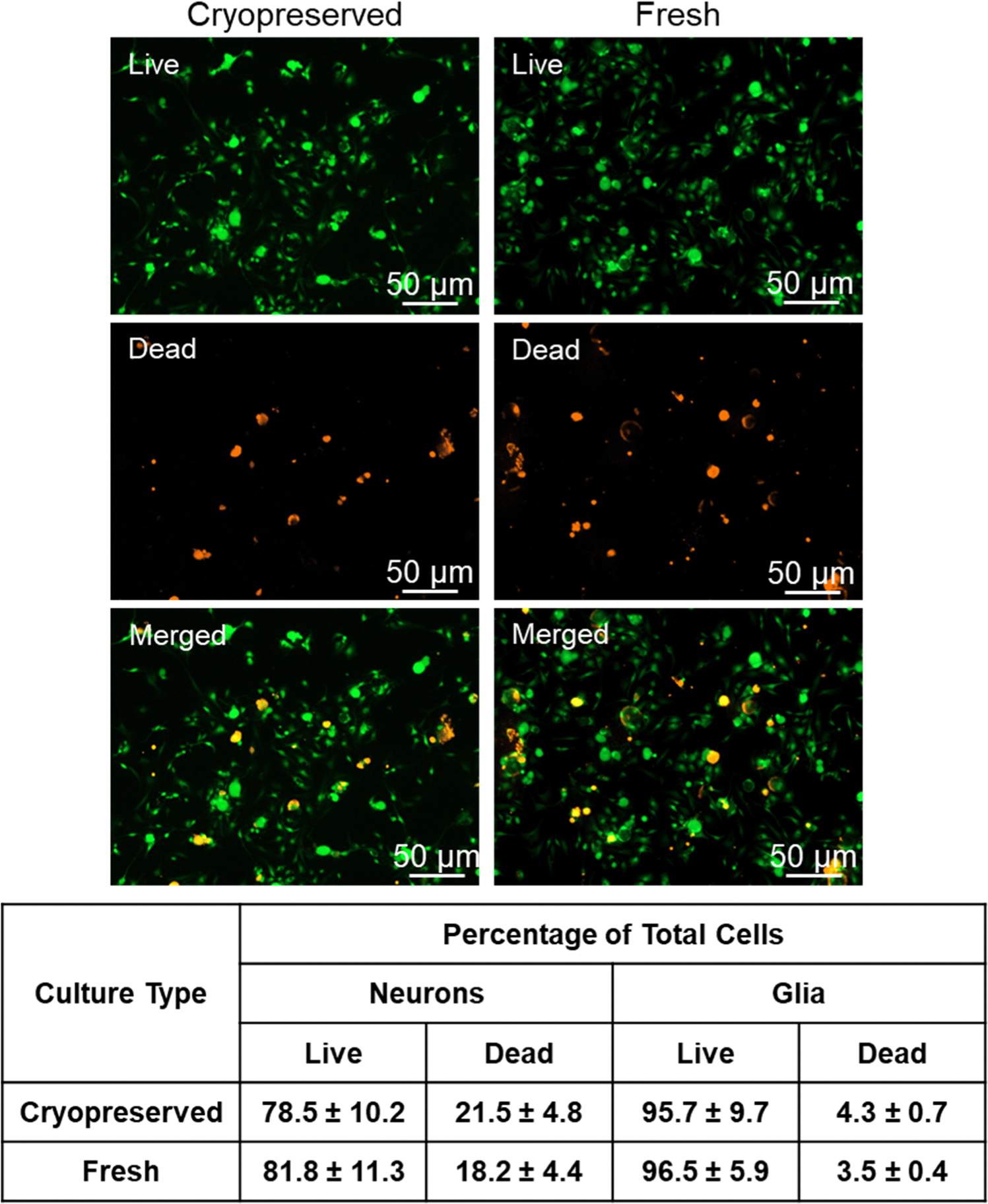
Comparison of cell viability of cryopreserved primary cultures to fresh preparation. Representative 100x fluorescent images from the LIVE/DEAD cell viability assay are shown for live (green), dead (red), and merged images. The percentage ± SEM of live or dead neurons or glia in cryopreserved and fresh cultures is shown in the table (n = 12 independent experiments performed in triplicate).

To determine cell viability after overnight incubation of cryopreserved and fresh cultures, a LIVE/DEAD assay was utilized to quantify the number of live versus dead cells. As seen in Fig. 2, the percentage of live vs dead cells were similar in the cryopreserved and fresh cultures. Cell counts from 3 images per coverslip were combined from 12 individual experiments for a total of 673 live neurons and 184 dead neurons, corresponding to an average of 78.5% of cells being viable, and 21.5% of total neurons being nonviable after cryopreservation (Fig. 2). Fresh cultures from 12 experiments were also analyzed in triplicate, for a total of 621 live neurons, and 138 dead neurons. Fresh cultures showed similar viability to cryopreserved cultures with 81.8% of neurons being alive, and 18.2% dead (p = 0.119, and p = 0.470 respectively). For glial cells, 4433 live and 199 dead cells were counted from cryopreserved preparations, and 3468 live and 125 dead glia were analyzed from fresh cells. There was no significant difference in the percentage of live glia between cryopreserved and fresh cultures (95.7%, 96.5%, p = 0.128), or between the percentage of dead glia (4.3%, 3.5%, p = 0.148).

### 3.3. Protein expression in cryopreserved neurons and glia

**Fig. 3.**
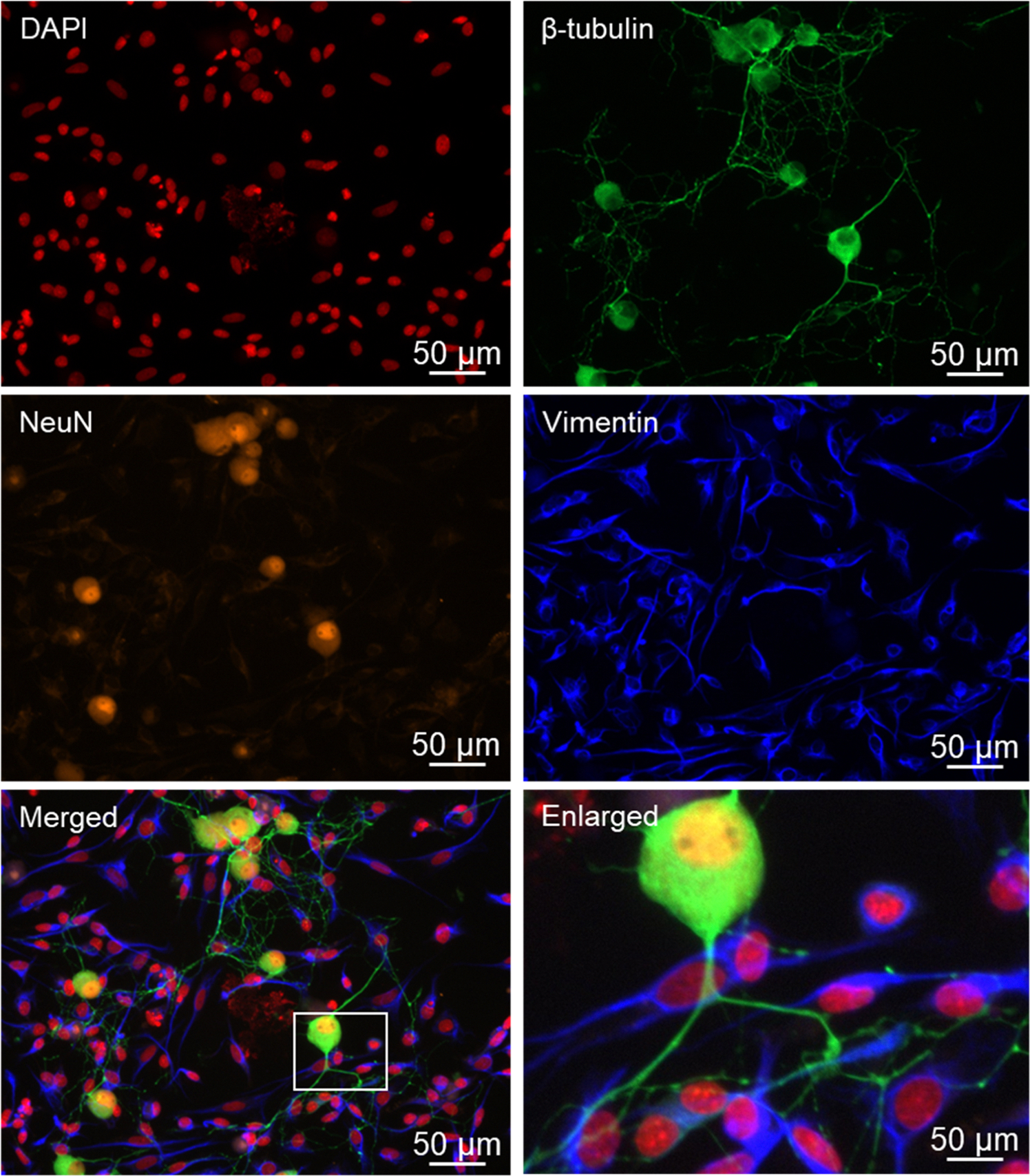
Primary cultures from cryopreserved trigeminal ganglion express neuronal and glial biomarkers. Overnight cultures were stained with the nuclear dye DAPI and the cytoskeletal protein β-tubulin, which was expressed in the cytoplasm and processes of neurons. The same culture was costained for NeuN, a protein biomarker of neuronal nuclei, and the cytoskeletal protein vimentin, which is a biomarker of glia and was expressed in satellite glial cells and Schwann cells. A merged image of all four individual channels (DAPI, β-tubulin, NeuN, and vimentin) is shown. An enlarged image, delineated by the white box in the merged channel image is shown that demonstrates the pseudo-unipolar morphology of a trigeminal neuron.

Initially cultures were co-immunostained with the nuclear dye DAPI and for neuronal biomarkers including the microtubule protein β-tubulin and the nuclear dye NeuN, or for the glial cell biomarker vimentin, which is an intermediate filament protein (Fig. 3). While β-tubulin was abundantly expressed in the cell body and processes of both Aδ and C fiber neuronal cell bodies, NeuN immunostaining was readily observed in the nucleus of both types of neurons. The pseudo-unipolar morphology of trigeminal neurons, which is characterized by a single process emanating from the cell body that then bifurcates into two branches, is clearly visible in the enlarged image. In addition, the length of the neuronal processes was similar between fresh (74.2 ± 5.8 μm) and cryopreserved (80.2 ± 4.2 μm). In glia, vimentin immunostaining was detected in the cytoplasm and processes of satellite glial cells and Schwann cells but not neuronal cells.

**Fig. 4.**
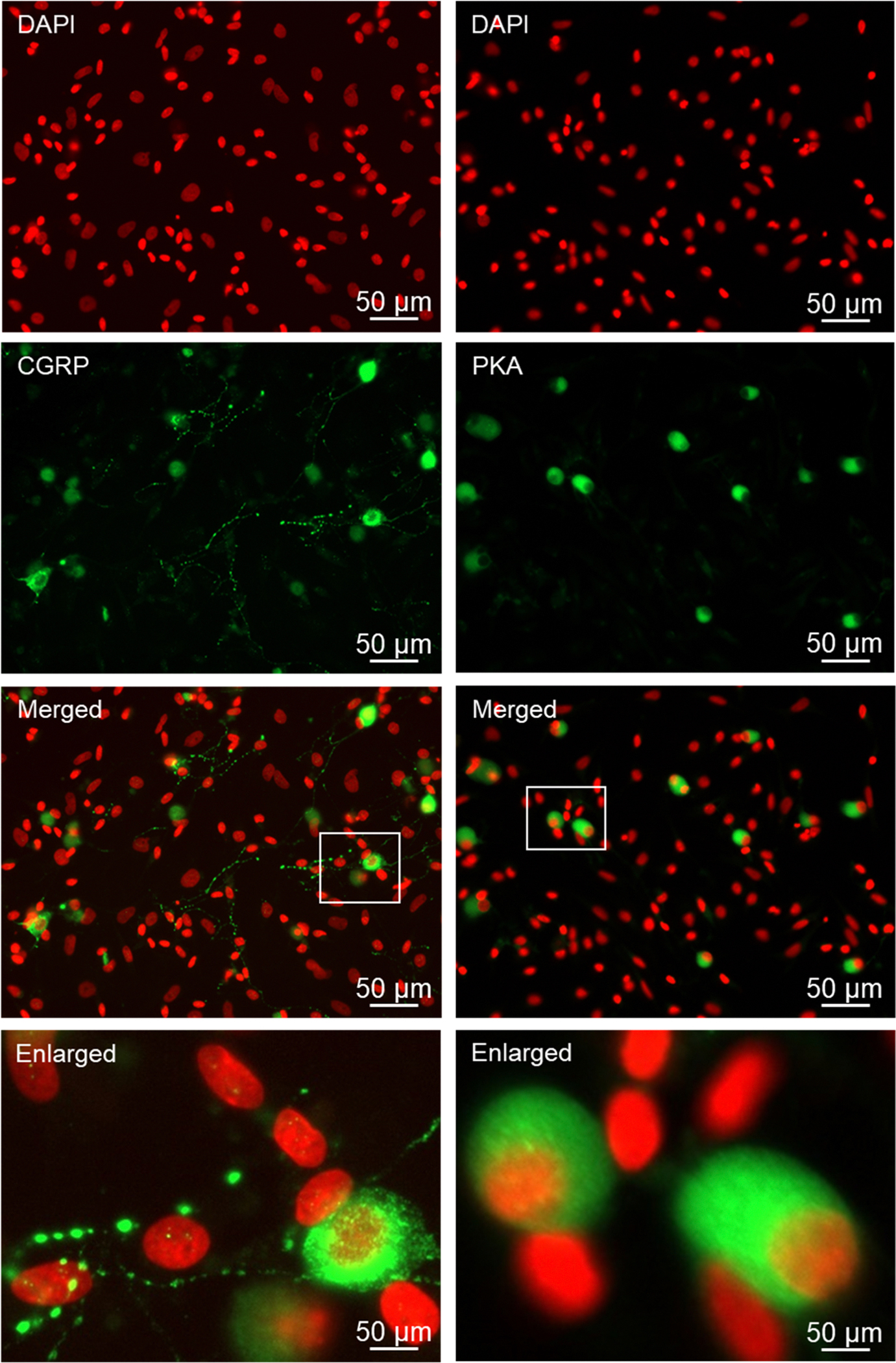
Cryopreserved primary cultures express the neuropeptide CGRP and signaling protein PKA. Primary cultures after overnight incubation were costained for DAPI and CGRP, or DAPI and PKA. Merged images and an enlarged area delineated by the white box are shown for each set of proteins.

As seen in Fig. 4, the neuropeptide CGRP was abundantly expressed in the cell body of both Aδ and C fiber neurons. CGRP immunostaining was also visible in the neuronal processes in a punctate pattern in unstimulated, basal culture conditions 24 h after plating. Immunostaining for the active form of the signaling protein PKA was readily observed in the cell body of Aδ and C fiber neurons. Neither CGRP nor PKA expression was detected in satellite glial cells or Schwann cells.

**Fig. 5.**
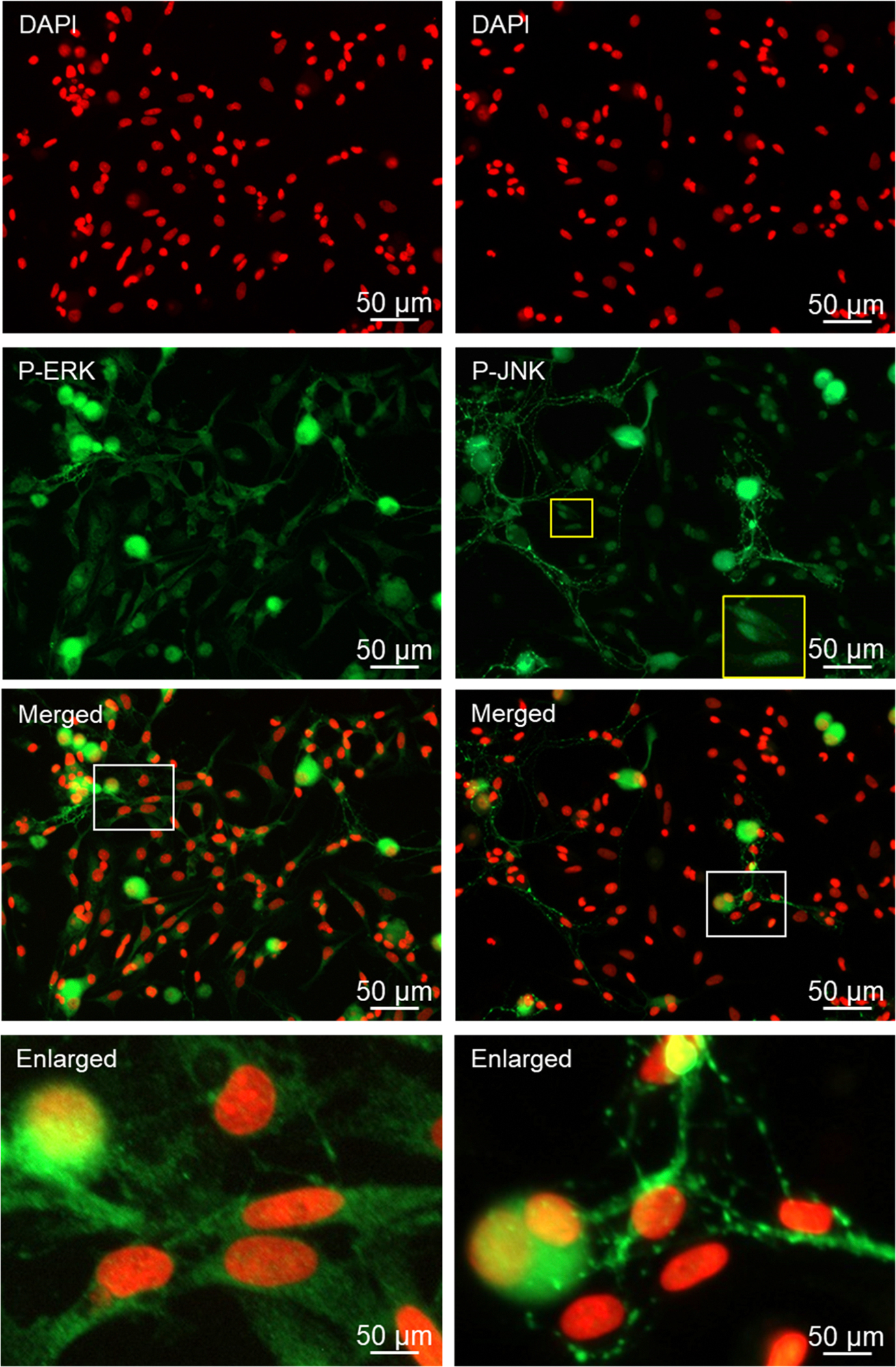
The active forms of P-ERK and P-JNK are expressed in cryopreserved neurons and glia. Primary cultures were costained for DAPI, P-ERK and DAPI, P-JNK. The yellow box in the P-JNK individual channel image indicates the glial cells enlarged in the bottom right corner of the image to show nuclear staining.

**Fig. 6.**
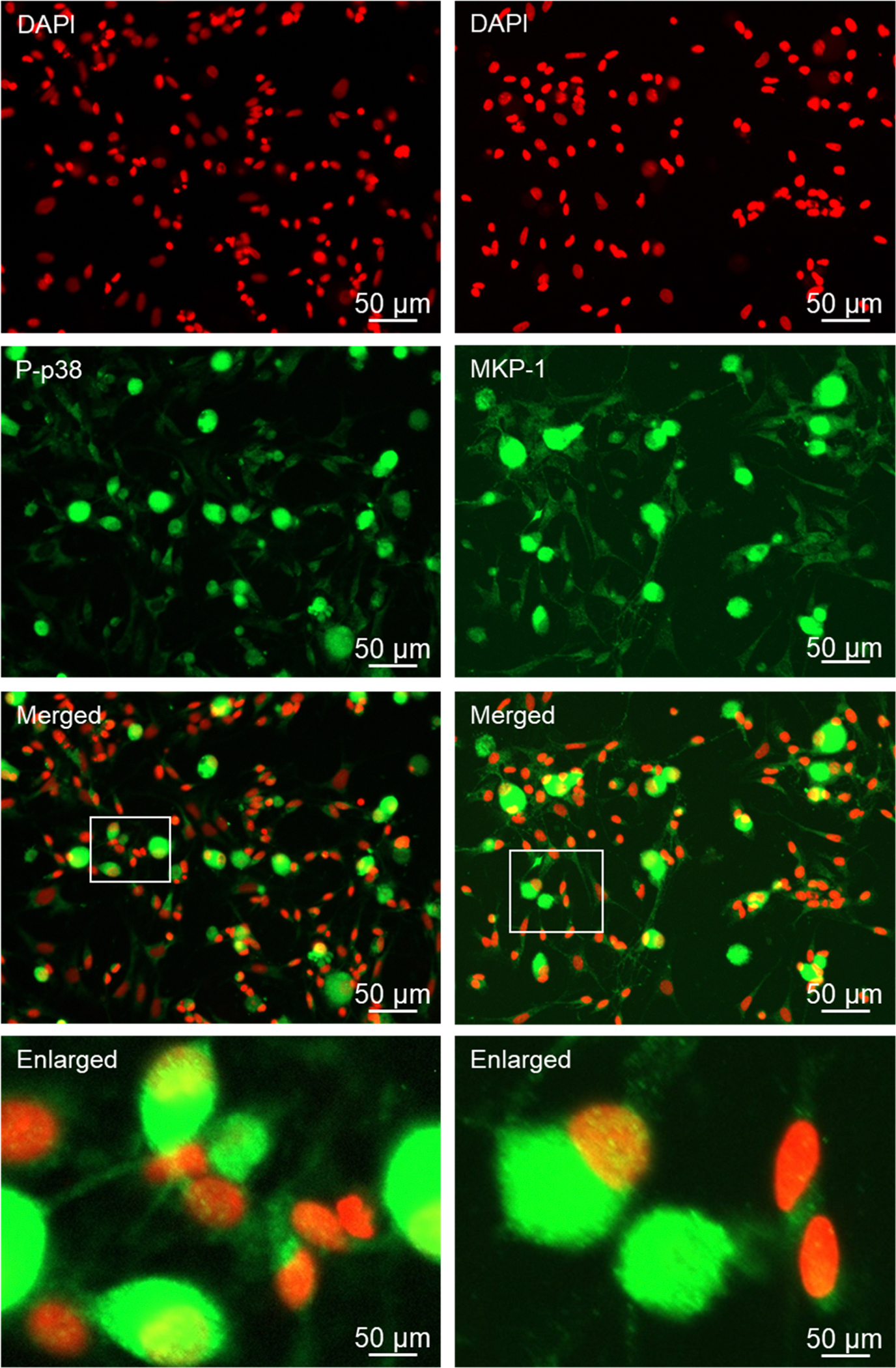
The active form of p38 (P-p38) and MAP kinase phosphate MKP-1 proteins are expressed in cryopreserved neurons and glia. Primary cultures stained for DAPI, P-p38 and DAPI, MKP-1 are shown. Enlarged images delineated by a white box in the merged channel images are presented on the bottom row for each protein.

The phosphorylated activated MAP kinase proteins P-ERK, P-JNK, and P-p38 were expressed in the cell body of most Aδ and C fiber neurons and the nucleus of many neurons under basal culture conditions (Fig. 5, Fig. 6). Although the intensity of immunostaining was less in glial cells for these proteins, each of the MAP kinases were expressed in the cytoplasm of most satellite glia and Schwann cells with nuclear staining observed in some glial cells under naïve, unstimulated conditions. A similar pattern was observed for the MAP kinase phosphatase MKP-1, which was expressed in the cytoplasm and nucleus of both neuronal cell types and glial cells, with immunostaining being more abundant in neurons than glia (Fig. 6).

**Fig. 7.**
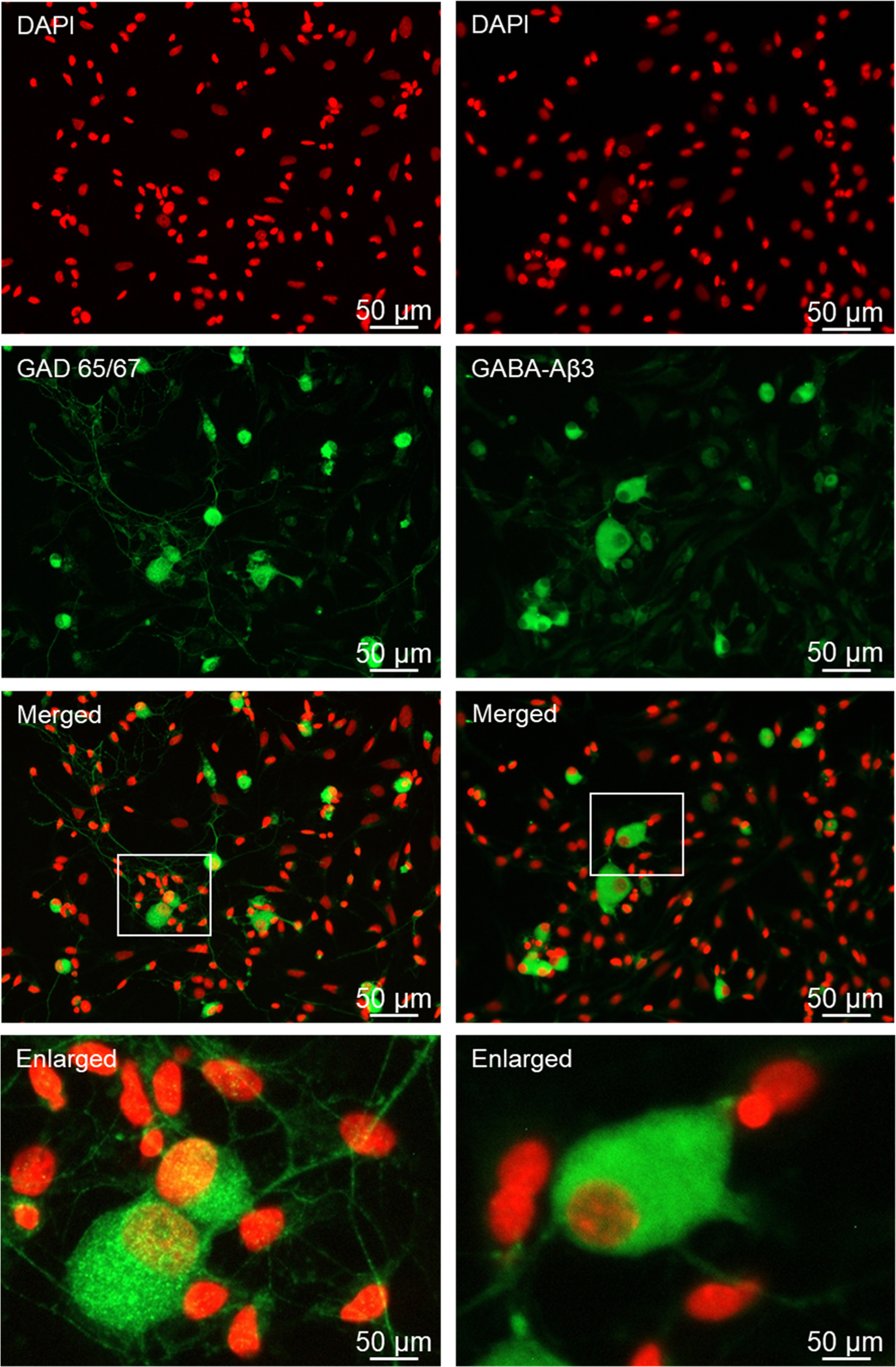
GABA synthesizing enzymes GAD 65/67 and GABAA receptor are primarily expressed in neurons of cryopreserved trigeminal ganglion cultures. Primary cultures stained for DAPI, GAD 65/67 and DAPI, GABAA are shown in individual channel images and merged images. Enlarged images are shown from the merged channel, as indicated by the white box.

**Fig. 8.**
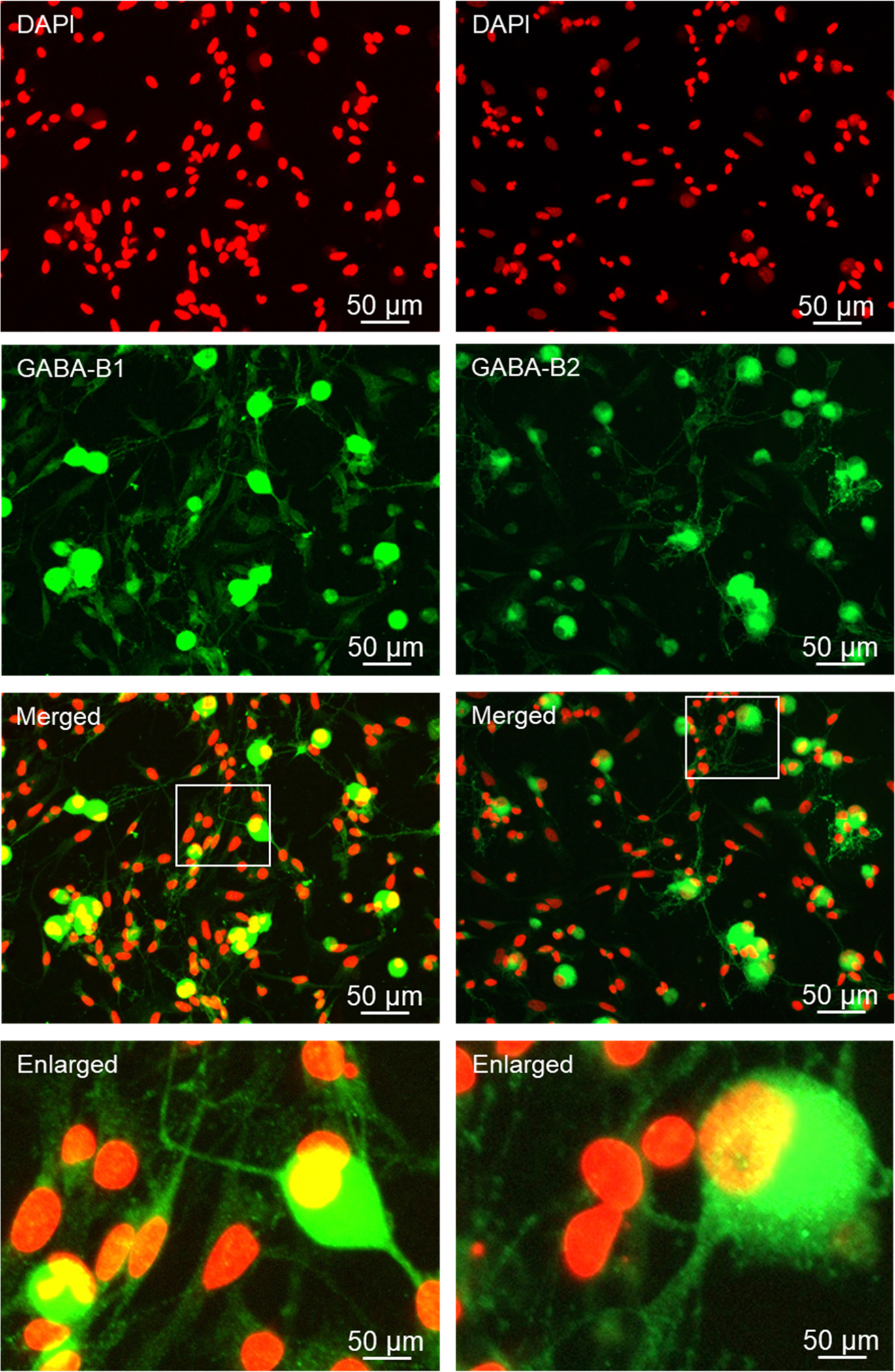
Neurons and glia abundantly express GABAB1 and GABAB2 receptor proteins. Primary cultures were stained for DAPI, GABAB1 and DAPI, GABAB2, with enlarged images underneath.

The GABA synthesizing enzymes GAD 65/67, as well as the GABA receptor subunit Aβ3 were readily detected in Aδ and C fiber neurons but not glial cells under basal conditions (Fig. 7). GAD 65/67 was expressed in the cytoplasm and processes of both types of neuronal cells. GABAA immunostaining was primarily associated with the cell body, but not the processes, of both types of neurons. In contrast, the GABAB receptor subunits, GABAB1 and GABAB2 were expressed in all four cell types, with the most abundant expression being observed in Aδ and C fiber neurons (Fig. 8).

### 3.4. Calcium imaging

**Fig. 9.**
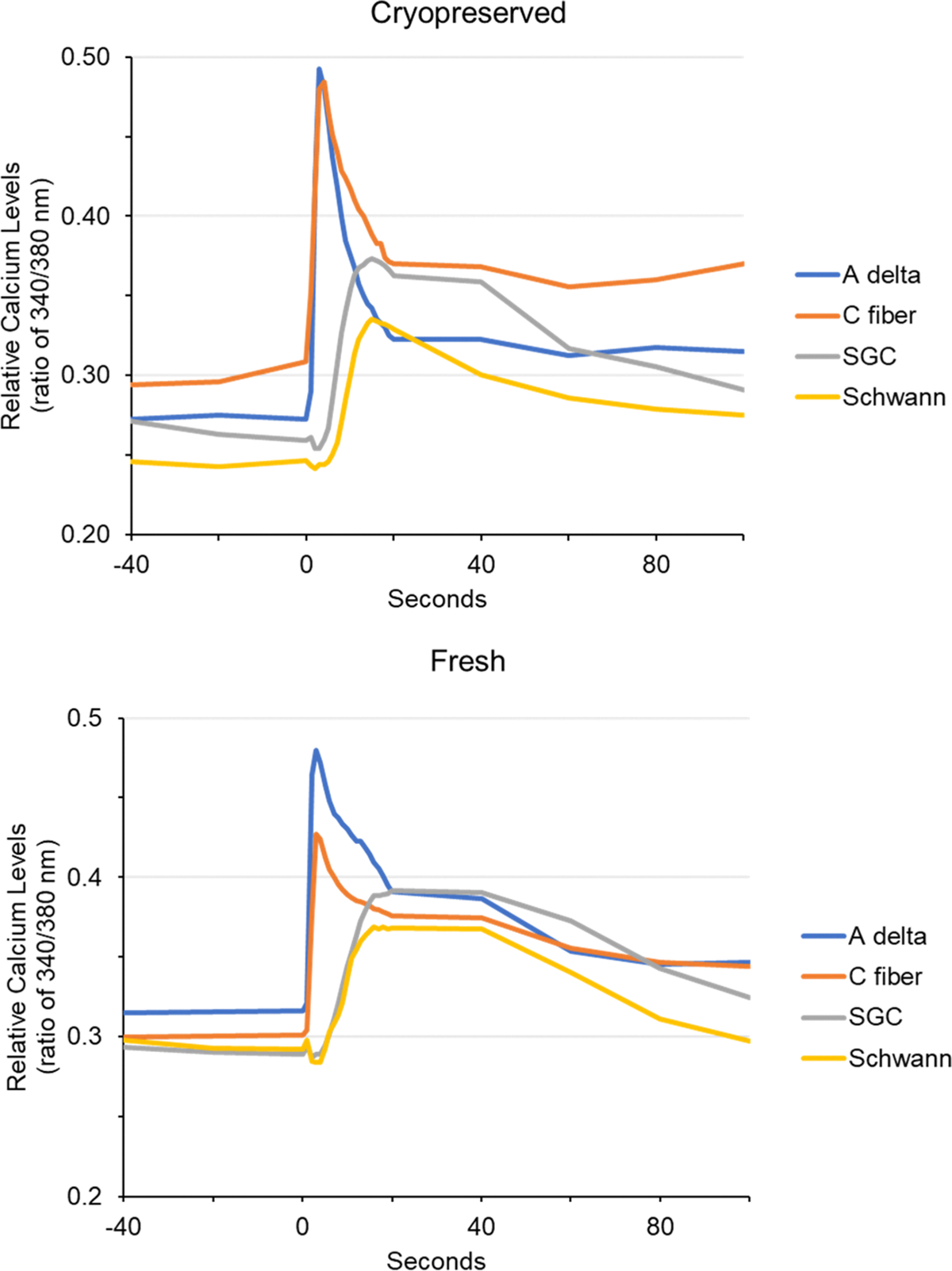
Calcium channels in neurons of cryopreserved and fresh culture preparations are stimulated in response to potassium chloride. A ratio of intracellular and extracellular calcium levels in neurons and glia of cryopreserved (top, n = 5) and fresh (bottom, n = 7) trigeminal ganglion cultures. Aδ and C fiber neurons (blue, orange) showed rapid stimulation. Satellite glial cells (gray) and Schwann cells (yellow) showed a smaller delayed and more sustained stimulation.

To demonstrate functional ion channel activity, the change in intracellular calcium levels in response to addition of KCl at a depolarizing concentration of 60 mM was determined using the ratiometric dye Fura-2 in cryopreserved and fresh primary culture preparations. In response to KCl, Aδ and C fiber neuronal cells exhibited a rapid transient increase in intracellular calcium levels (Fig. 9). A delayed and more sustained increase in intracellular calcium was observed in satellite glial cells and Schwann cells in response to KCl stimulation. A similar pattern of calcium response to KCl was observed in cryopreserved and fresh primary trigeminal ganglion cultures.

### 3.5. Transient transfection

To demonstrate that cryopreserved trigeminal ganglion primary cultures can be successfully transfected to study changes in transcription, cells were transiently cotransfected with a plasmid construct with the CMV promoter and luciferase gene and CMV promoter with the β-galactosidase gene. The average basal β-galactosidase activity was 317,992 relative light units when normalized to total protein per well while the average basal normalized luciferase activity was 86,638 relative luciferase light units per well (Table 3). These results demonstrate that cryopreserved trigeminal ganglion cultures can be successfully transfected when using a similar protocol to previous published studies that utilized fresh preparations of trigeminal ganglion cultures (Durham and Russo, 1999, 2003, 1998).

## 4. Discussion

The utilization of primary cell cultures provides an important model for understanding biochemical, cellular, and molecular mechanisms involved in pathological and therapeutic processes prior to conducting more expensive and time-consuming *in vivo* studies. For example, this approach was used to understand the cellular and molecular mechanisms by which biologically active compounds in cocoa inhibited trigeminal neuron activation *in vitro* and suppressed inflammation and pain signaling *in vivo* (Abbey et al., 2008; Cady and Durham, 2010; Cady et al., 2013). However, in those initial studies, fresh cultures of trigeminal ganglia were utilized to generate the *in vitro* data which greatly added to the overall cost and time required. In this study, we have optimized the conditions for cryopreservation of trigeminal ganglia obtained from 4 to 5 day old Sprague-Dawley neonatal rats such that the viability, morphology, neuron-glia ratios, and function are maintained even after one year of storage. While other commercial and published protocols typically use culture media supplemented with a lower percentage of FBS with either 5 or 10% DMSO (Seggio et al., 2008; Parker et al., 2018; Ishizuka and Bramham, 2020; Cui et al., 2021; Lin et al., 2023; Valdor et al., 2018), our cryopreservation medium consisted of 90% FBS and 10% DMSO as the cryoprotectant. We acknowledge that there are likely other factors besides the utilization of a 90/10 FBS: DMSO medium that contributed to the high viability and preservation of cell function observed in our mixed neuron/glia cultures including how the cells where frozen and thawed and the temperature of the medium at each step in the process. On the day of the experiment, cryopreserved cells were slowly thawed, and the freezing media diluted with a large volume of 37 °C plating media prior to centrifugation. Cells were plated on poly-D-lysine coated coverslips or wells, which maintained the viability and morphology of both neurons and glial cells. In contrast to other studies that used their ganglion cultures after storage for only weeks or months (Seggio et al., 2008; Cui et al., 2021), the viability, morphology, protein expression, and calcium response was preserved even after storage for more than one year. Similarly, primary cultures of rat hippocampal and cortical neurons prepared using a 45% FBS:10% DMSO cryopreservation medium were reported to maintain good viability and were developmentally and functionally similar to fresh prepared cultures upon thawing after one year of storage in liquid nitrogen (Ishizuka and Bramham, 2020). A recent study reported a method for cryopreservation of trigeminal ganglion based on the method of Ishizuka and Bramhan (Ishizuka and Bramham, 2020) that was used for enrichment of neuronal cells that provide sensory innervation of the cornea for studying the effects of drugs and neurotoxins (Lin et al., 2023). In both of those studies, the objective was to establish neuronally-enriched cultures devoid of glial cells. The goal of our study was to establish trigeminal ganglion cultures of neurons and glial cells in the ratio present in the ganglion of 3–5 day old neonatal animals. The simple method described in our study allows for long-term storage of trigeminal ganglion neurons and glial cells that maintain their viability, morphology, and function and thus could be useful for conducting *in vitro* studies.

Following overnight incubation of the cryopreserved trigeminal ganglion on poly-D-lysine coated glass coverslips, Aδ and C fiber neurons exhibited healthy morphology as evidenced by a round cell body, nuclear localization of the neurofilament protein NeuN, which is a biomarker of post-mitotic neurons, and extensive neuronal processes expressing the structural microtubule subunit β-tubulin. In some instances, the neuronal cells exhibited a pseudo-unipolar morphology characteristic of ganglionic sensory neurons (Messlinger et al., 2020; Zenker and Hogl, 1976). Under our culture conditions, both Aδ and C fiber neurons showed similar viability and as expected, the percentage of C fiber neurons was much greater than those of Aδ, a finding in agreement with the ratios observed in cross sections of trigeminal ganglion (Thalakoti et al., 2007). Our cryopreservation technique also maintained the viability and unique morphology of the satellite glial cells and bipolar Schwann cells. Both glial cells expressed the structural intermediate filament protein vimentin, which is used as a biomarker of active glial cells, in the cytoplasm and processes. Importantly, cryopreserved trigeminal ganglion cultures express proteins that cause the development and maintenance of peripheral sensitization and pain signaling in orofacial pain diseases including migraine and TMD. The neuropeptide CGRP, which promotes neurogenic inflammation and enhances pain signaling (Romero-Reyes et al., 2015; Greco et al., 2022), was abundantly expressed in the cytoplasm and neurites as axonal varicosities (bouton) of both Aδ and C fiber neurons under basal, unstimulated culture conditions. Activation of the CGRP receptor is known to cause activation of the pro-inflammatory protein PKA and the MAP kinases in the trigeminal ganglion (Sun et al., 2004; Cady et al., 2011; Cornelison et al., 2016; Nie et al., 2022; Vause and Durham, 2009). PKA immunostaining was readily observed in the cytoplasm and nucleus of both types of neurons as well as at a lower level of expression in the cytoplasm and nucleus of satellite glia and Schwann cells. Similarly, cytoplasmic and nuclear expression of P-ERK and P-p38 were detected in both types of neurons and glial cells. In contrast, P-JNK expression was observed more abundantly in neuronal cells in the cytoplasm and nucleus, and a punctate pattern in the neurites indicative of axon varicosities that was similar to CGRP. The MAP kinase phosphatase MKP-1, which functions to inactivate the MAP kinases and restore and maintain cellular homeostasis (Owens and Keyse, 2007), was expressed in a pattern similar to P-ERK and P-p38 with immunostaining detected primarily in the cytoplasm of both types of neurons and glial cells. Another pathway that is known to inhibit neuronal and glial cell activation involves the GABAergic proteins including GAD 65/67 that is responsible for the synthesis of the inhibitory neurotransmitter GABA and the GABAA and GABAB receptors (Lee et al., 2019). In our study, GAD 65/67 was expressed in the cytoplasm of both Aδ and C fiber neurons but not glia, a finding in agreement with results from a recent *in vitro* study of trigeminal ganglion (Antonopoulos and Durham, 2022). However, immunostaining for GABAA and GABAB receptors was observed in both types of neurons but also associated with satellite glia and Schwann cells. Taken together our findings provide evidence that our method for cryopreservation and culturing of trigeminal ganglion cells maintains the normal expression pattern of key proteins implicated in inflammation and pain signaling in diseases involving sensitization and activation of trigeminal neurons and glia.

Another major finding was that cryopreserved trigeminal neurons and glial cells respond similarly to a depolarizing stimulus as compared to freshly prepared trigeminal ganglion cultures. Addition of KCl to the culture media caused a rapid, transient increase in the level of intracellular calcium in both Aδ and C fiber neurons while mediating a delayed, less robust transient increase in satellite glia and Schwann cells. These results provide evidence that the cryopreserved cultures can be utilized to study changes in calcium ion channel activity in response to different stimuli or inhibitory agents. While the focus of this study was to determine changes in intracellular calcium, the cultures could be used to investigate the role of specific ion channels, such as sodium or chloride, under different experimental conditions that simulate disease pathology or therapeutic mechanisms. Another potential use of cryopreserved trigeminal ganglion might involve the study of transcriptional activity as determined following transient transfection of reporter genes or viral infection. Under basal, unstimulated culture conditions, both luciferase and β-galactosidase activity were detected following transient transfection and hence this method could be utilized for study of promoter activity as done previously using fresh prepared cultures of trigeminal ganglion (Durham and Russo, 1999, 2003). Our findings agree with the results of the study by Seggio et al. (2008) that provided evidence of successful cryopreservation of primary transfected and non-transfected dorsal root ganglion neurons and the study by Valdor et al. (2018) in which similar gene expression was reported for adeno-associated viral vector mediated transduction of fresh and cryopreserved neuronal cultures of dorsal root ganglion. In summary, the demonstration that cryopreserved trigeminal ganglion cells exhibit functional calcium ion channel activity in response to a depolarizing stimulus and CMV promoter reporter gene expression provides evidence of maintenance of key cellular functions.

The goal of this study was to develop an efficient and reliable method for establishing primary neuron/glia mixed cultures of trigeminal ganglion for biochemical, cellular, and molecular studies that may involve toxicity, protein expression, calcium ion imaging, and gene expression. Advantages of using cryopreserved cells include providing greater flexibility in planning experiments, resource and cost savings since requires fewer preparations, and allowing an investigator to share the cryopreserved cells with other laboratories to facilitate validation and collaboration of research findings. Importantly, our technique preserved the viability, morphology, expression of proteins implicated in inflammation and pain signaling, functional calcium ion channel activity, and reported gene expression in neurons and glial cells. A unique difference with our technique involved the use of a 90% FBS/10% DMSO freezing medium that provided a reliable technique for long-term cryopreservation of trigeminal ganglion primary cultures established from neonatal Sprague Dawley rats that would increase research productivity. While the protocol described in this study was used for establishing mixed cultures of trigeminal ganglion that approximate the number of neurons and glia as seen *in vivo* (Thalakoti et al., 2007), enrichment for either neuronal or glial cells could be achieved by using a BSA or Percoll gradient or similar method (Vause and Durham, 2010; Abbey et al., 2008; Li et al., 2008). Given the evidence of using this method for cryopreserving trigeminal ganglion, it is highly likely that this technique could be useful in the cryopreservation of dorsal root and nodose ganglia, and central nervous system tissues including specific regions of the spinal cord and brain. In conclusion, our method provides a straightforward and reliable procedure that can be readily implemented as a standard laboratory practice for long-term storage of viable, healthy trigeminal ganglion neurons and glia.

## Figures and Tables

**Table 1:** Information on primary antibodies used in this study.

Protein	Company	Product Number	Dilution

β-Tubulin	Abcam	ab18207	1:5000
NeuN	Sigma-Aldrich	MAB377	1:1000
Vimentin	Thermo Fisher Scientific	PA5-142829	1:5000
CGRP	Sigma-Aldrich	C8198	1:2000
PKA	Abcam	ab76238	1:5000
P-ERK	Neuromics	RA15002	1:2000
P-JNK	Abcam	ab47337-1001	1:5000
P-p38	Abcam	ab4822	1:5000
MKP-1	Abcam	ab61201	1:2000
GAD 65/67	Abcam	ab11070-1001	1:5000
GABA-Aβ3	Abcam	ab98969-1001	1:5000
GABA-B1	Abcam	ab55051-1001	1:5000
GABA-B2	Abcam	ab75838	1:5000

**Table 2:** Characterization of cell types in cryopreserved and fresh primary cultures (n = 5).

	Cryopreserved	Fresh

**Neurons**	12.3%	10.4%
Aδ Fiber	2.4%	2.4%
C Fiber	9.9%	8.0%
**Glia**	87.7%	89.6%
Satellite Glia	61.2%	59.5%
Schwann Cells	26.5%	30.1%

**Table 3:** Cryopreserved neurons and glia show reporter gene activity. Cultures were transiently transfected with the reporter genes β-galactosidase and luciferase. Results are reported as light units ± SEM normalized to the total protein per well (n = 4).

Reporter Gene	β-galactosidase (Light Units)	Luciferase (Light Units)

N = 4	317,992 ± 26,836	86,638 ± 12,948

## Data Availability

Data will be made available on request.
